# Woman's Help for Woman

**Published:** 1889-03-02

**Authors:** 


### The Hospital

A WEEKLY INSTITUTIONAL JOURNAL OF

**Science, Medicine, Nursing, and Philanthropy.**

*For Hospitals, Asylums, Medical Practitioners, Students, Nurses, Families, Parishes, Congregations, and the Charitable Public.*

VOL. V. No. 127.] [MARCH 2, 1889.

### WOMAN'S HELP FOR WOMAN.

WOMEN have long been claiming the freedom to exercise certain rights; men are now beginning to discover the superior fitness of women for certain duties. If both men and women preserve an open mind there will be a gradual shaking down during the next few years, which will result in a wiser appropriation of duties to both sexes than has been common in past times. What fact could be more strange and surprising than one incidentally brought to light in the columns of the *Times* the other day by Mr. William Tallack, of the Howard Association? Mr. Tallack writes to say that the State of New York has passed an Act empowering the Mayor or Police Commissioners of every town of twenty-five thousand inhabitants or upwards to "designate one or more station-houses for the detention and confinement of all women under arrest," and that "at least one such station-house shall at all times be so designated in each city." The Act further prescribes that "no woman shall be appointed a police matron unless suitable for the position and recommended therefor in writing by at least twenty women of good standing, residents of the city in which the appointment is made." One such matron is always to be in readiness for duty day and night. The Act also authorises the appropriation of funds needful to provide "sufficient accommodation for women held under arrest, to keep them separate and apart from the cells, corridors, and apartments provided for males held under arrest."

There is nothing surprising in the fact that the State of New York has provided for women to be kept separate from men in public prisons, and that in each women's prison there shall be a wholesome and well-conditioned matron, who shall have practical supervision over the members of her own sex. What is surprising, even to astonishment, is that so evident a necessity as separate places of detention and matronly supervision should not have been in use for women all the world over during the last eighteen hundred or two thousand years. For incorrigible prisoners of both sexes adequate severity of punishment is indispensable. But all criminals are not of this class, and especially, it may be hoped, not all women criminals. Undoubtedly, even among women, there are some whose moral sense is so feeble, whose intelligence is so deficient, and whose passion for self-indulgence is so strong that no law, human or divine, can either instruct them or make them afraid. But surely these are the exceptions! In ordinary women, even of the criminal class, there is a gentleness of nature, an innate sense of decency and good behaviour, a love for the safety and protection which goodness brings with it, that if properly worked upon would lift many a degraded woman into purity, honesty, and wholesomeness of life.

It is here where women are so all-potent with women. How can a woman, who is in prison for the first time, lay bare her sorrows and her sins to a male attendant or a male governor? She probably never even thinks of it. The governor or the attendant, on the other hand, has neither the knowledge nor the aptitude to address himself directly to the woman's heart and better nature. The difference of sex is like a wall built round both prisoner and guard, and each looks upon the other as an enemy and a danger. But a woman of experience and gentle nature, a woman of firm and controlling will, can see clearly into the depths of another woman's soul, and can show such knowledge and sympathy as will put to the blush all that is worst in her fellow-woman, and kindle into life the few smouldering embers of virtue and hope which may yet be left in her heart. A police matron must not of course be a she-dragon or a namby-pamby sentimentalist who will be cheated by even the babes in crime. The State of New York has provided against that in an eminently reasonable and practical way. It has in effect placed the appointment in the hands of women. "No woman shall be appointed as police matron," runs the statute, "unless suitable for the post and recommended therefor, in writing, by at least twenty women of good standing, residents of the city in which the appointment is made." There is in this provision an excellent guarantee that as a rule suitable women will be appointed. There need, therefore, be no anxiety whatever as to the result of the new departure. The thing is in itself so obviously reasonable, so completely in agreement with all that is natural and right and kind that the public may be absolutely certain of marked changes and improvements among the female criminals of the New York State.

A comparison of the condition of affairs at home with that which obtains in New York State does not redound to the credit of British prison administrators. It is true that a Home Office Commission has sat upon the court-houses and police-stations of England, and that material improvements both in structure and administration may be expected. It is also true that under Mr. Matthews' régime the employment and payment of female officers at the police courts has been approved and sanctioned, and that steps are now being taken to provide competent attendants. That seems to be as far as we have gone in this direction in England. There is no need to ask whether or not it is far enough. It is not far enough. In ordinary legislation it is perhaps wise to hasten slowly. But in cases where opinions can hardly be divergent, where reason, experience, and good feeling all concur in pointing out a new departure, that departure should be made with all possible promptitude and thoroughness. It is an unhappy thing that any civilised state should have a large criminal population; it ought to be a grief and a shame that a considerable proportion of that population consists of women and girls; it should be held a disgrace that anything is left undone which can diminish the number of women and girls who belong to the vicious and criminal ranks. That disgrace must be ours so long as we neglect to provide for every prison, poor-house, or other place of detention where vicious and criminal women are held in custody, suitable matrons and female attendants who may do what is possible to help their sister women back to virtue. There are no sources of information available whereby we may state exactly the number of police matrons and female attendants now employed in this country, and the number actually needed for the proper carrying out of the work of reclamation among women. It may, however, be taken for granted that we are yet in the very earliest stages of this great movement of prison reform and female reclamation. So far as we have advanced the results are more than satisfactory. It is found that in various directions, in regard both to prostitutes and female criminals, female officers are not only equal, but decidedly superior to males in efficiency. This fact has long been recognised and acted upon to some extent by prison authorities, but it "requires to be similarly and thoroughly adopted in the police and detective departments."

Practical administrators should recognise that here, if nowhere else, women are undoubtedly superior to men. In urging a plea for their immediate and universal employment we are not putting forward an argument for virtuous women's rights, but for fallen women's wrongs. The men of this country are not pharisaical. They do not consider a lapsed woman a criminal so vicious that she is not worthy of a single saving effort. On the contrary, there is a great deal of patience and forbearance with the women who have sunk below the level of virtue, and a very deep and real desire to bring them if possible back to decent ways. But that feeling should be put into practical shape and force. Needed reforms cannot be left to official minds. When did an official ever see anything but what was perfect in his own department? Philanthropists and statesmen must inform themselves on the subject; they must take it seriously to heart; they must bring conscience as well as intelligence to bear upon it; and when they have satisfied their minds of the necessity and value of female help in the reclamation of vicious and criminal women, they must let no grass grow under their feet until every institution in the land which needs such help is thoroughly and abundantly supplied.

